# Amplifying and ameliorating light avoidance in mice with photoreceptor targeting and calcitonin gene‐related peptide sensitization

**DOI:** 10.1111/head.70018

**Published:** 2025-12-15

**Authors:** Eric A. Kaiser, Audrey Cavanah, Geoffrey K. Aguirre, Frances E. Jensen

**Affiliations:** ^1^ Department of Neurology University of Pennsylvania Philadelphia Pennsylvania USA

**Keywords:** calcitonin gene‐related peptide, intrinsically photosensitive retinal ganglion cells, melanopsin, migraine, photophobia, photoreceptors

## Abstract

**Objective:**

The aim of this study was to determine the photoreceptor basis of light avoidance in mice and assess the effect of CGRP sensitization on this behavior.

**Background:**

Prior studies have suggested that photophobia is mediated by a subset of retinal ganglion cells (RGCs) that contain melanopsin, making them intrinsically photosensitive (ipRGCs). These cells also receive extrinsic input from cones, which can also mediate light sensitivity. Here, we examined how spectral targeting of melanopsin or specific cone types in mice produces light avoidance and whether sensitizing mice with calcitonin gene‐related peptide (CGRP) amplifies the avoidance response to ipRGC stimulation.

**Methods:**

Light avoidance behavior was measured in a two‐zone chamber illuminated by narrow‐band light‐emitting diodes (LEDs) targeting photopic opsins: 365 nm (ultraviolet [UV]; rodent S‐cone), 460 nm (blue; melanopsin), and 630 nm (red; human L‐cone). In a non‐targeted assay, we assessed the degree of light avoidance in wild‐type (WT) C57BL/6J mice to varying contrasts (0.05 to 1.00) of the blue and red LEDs. In a targeted assay, mice were exposed to zones with differing relative contrast levels (0.50, 0.75, or 1.00) for the targeted photoreceptor(s). This was assessed in transgenic mice with: (1) human L‐cone cone knock‐in (HLCKI) or (2) adult‐onset ablation of M1 ipRGCs (Opn4^aDTA^ *).* Mice were studied without intervention or following chronic intermittent administration of CGRP with either peripheral CGRP or vehicle (Veh) administration every‐other‐day for 9 days. A primary measure (mean +/− SEM) was the asymptote value (AV) of chamber preference.

**Results:**

WT mice showed greater light avoidance with increasing light contrast. HLCKI mice avoided zones with high melanopsin (1.00: 0.52 ± 0.08; *n* = 18) and L‐cone (1.00: 0.30 ± 0.11; *n* = 15) stimulation but showed a preference for the zone with higher S‐cone (1.00: −0.35 ± 0.06; *n* = 16) stimulation. These effects were contrast‐dependent. The addition of S‐cone stimulation reduced the aversive effect of melanopsin (0.10 ± 0.12; *n* = 14) or L‐cone (−0.19 ± 0.10; *n* = 15) contrast. Ablation of ipRGCs in HLCKI x Opn4^aDT*A*^ mice eliminated both avoidance of melanopsin stimulation and the preference for S‐cone stimulation, as compared to controls. Nine days of chronic intermittent administration of CGRP led to significantly increased avoidance of melanopsin stimulation (0.58 ± 0.08, *n* = 21) as compared to Veh administration (0.26 ± 0.09, *n* = 22) (*F* (1, 41) = 5.70, *p* = 0.022).

**Conclusions:**

Our findings support a key role for the ipRGCs in the production of photophobia. This aversive response to light stems from integrated ipRGC signals that combine excitatory intrinsic melanopsin and extrinsic L‐cone inputs and are opposed by extrinsic, inhibitory S‐cone input. Chronic elevation of CGRP levels in migraine may amplify ipRGC signals, leading to photophobia.

## Plain Language Summary

To better understand light sensitivity, a common symptom associated with migraine, we studied which cells in the eye produce the signals that cause mice to avoid light. We found that mice avoided blue and red light but preferred ultraviolet (UV) light, which is a result of a special cell type (intrinsically photosensitive retinal ganglion cells [ipRGCs]) in the eye that processes these light signals. We also found that mice who were repeatedly exposed to calcitonin gene‐related peptide (CGRP), a key nervous system messenger in migraine, increased their avoidance of blue light, which may model what happens in people with chronic migraine who experience light sensitivity.

**Abbreviations**
ANOVA: analysis of variance
AV: asymptote value
CGRP: calcitonin gene‐related peptide
HLCKI: human L‐cone knock‐in
ip: intraperitoneal
ipRGC: intrinsically photosensitive retinal ganglion cell
L: long wavelength
LED: light‐emitting diode
M: medium wavelength
Mel: melanopsin
RGC: retinal ganglion cell
S: short wavelength
UV: ultraviolet
Veh: vehicle
WT: wild‐type

## INTRODUCTION

**FIGURE 1:**
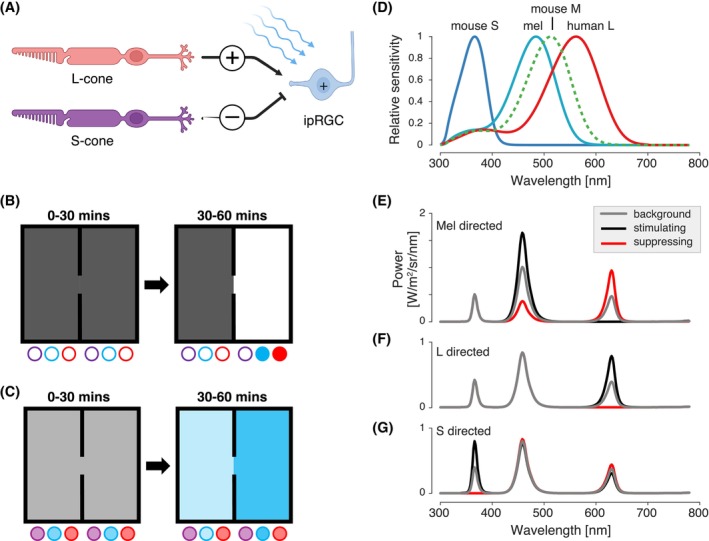
Experimental overview. (A) Schematic of the retinal network for transgenic human L‐cone knock‐in (HLCKI) mice, in which L‐cone stimulation provides excitatory, extrinsic input to ipRGCs, and S‐cones stimulation provides inhibitory, extrinsic input. Melanopsin (Mel) stimulation provides excitatory, intrinsic input to intrinsically photosensitive retinal ganglion cells (ipRGCs), which integrate these three signals. Created with Biorender.com. (B) In the non‐targeted behavioral assay, animals were placed in the two‐zone box. From 0 to 30 min, light‐emitting diodes (LEDs) were off in both zones. From 30 to 60 min, LEDs remained off in one zone, while the ultraviolet (UV), blue, and red LEDs were turned on in the second zone. Note that the UV LED was unintentionally blocked by a diffusing panel. The contrast level of the LEDs was set to 0.05, 0.15, 0.25, 0.50, and 1.00 of their maximal setting. (C) In the photoreceptor‐targeted behavioral assay, animals were placed in the two‐zone box. From 0 to 30 min, the LED settings in the two zones were changed to create a difference in contrast upon targeted photoreceptor(s) between the two chambers. The LED settings were scaled so that this stimulus produced 0.50, 0.75, or 1.00 of the maximal contrast available on the targeted photoreceptors. (D) The spectral sensitivity functions of the relevant photoreceptors under daylight conditions, including mouse S‐cone (blue), Mel (teal), mouse M‐cone, and human L‐cone (red). (E‐G) Three sets of spectral modulations (background, gray; low suppressing, red; stimulating, black) used for the photoreceptor‐targeted behavior assay. The photoreceptors targeted here include (E) mouse S‐cones, (F) Mel, and (G) human L‐cones. [Color figure can be viewed at wileyonlinelibrary.com]

Bright light can be perceived as uncomfortable, and discomfort from light is increased in numerous ophthalmologic and neurologic conditions, most notably migraine headaches. Light sensation begins in the retina, with phototransduction under daylight conditions chiefly by the cones and melanopsin (Mel). Humans are trichromats, possessing three cone classes (L, M, and S), corresponding to “long” (red, λ_max_ ≈ 560), “medium” (green, λ_max_ ≈ 530), and “short” (blue, λ_max_ ≈ 420) wavelengths, respectively; whereas, mice are dichromats, possessing two cone classes (M and S), corresponding to “medium” (green, λ_max_ = 511) and “short” (ultraviolet [UV], λ_max_ = 359) wavelengths, respectively (Figure 1A).^1^ Additionally, a subset of retinal ganglion cells (RGCs) contains the photopigment Mel, rendering them intrinsically photosensitive (ipRGCs), with peak sensitivity to cyan wavelengths (λ_max_ ≈ 480) (Figure 1A).^2^, ^3^ M1 ipRGCs, one of six subtypes, are responsible for non‐image‐forming responses to light, including circadian photoentrainment, and the pupillary light reflex.^4^ Like the “classical” RGCs that do not express Mel, the ipRGCs (especially the M1 class) also receive extrinsic inputs from the cones.^4^ A topic of general interest is how these different retinal signals contribute to a sense of visual discomfort.

Prior work in rodents suggests that the ipRGCs play a central role in the discomfort response. When exposed to blue light, neonatal mice (which have yet to develop functional rods and cones but have functional ipRGCs at birth) exhibit avoidance behaviors^5^ and produce negative vocalizations.^6^ Adult mice show a preference for dark spaces compared to brightly lit ones,^7^ and inactivation of the ipRGCs has been shown to attenuate this preference.^8^, ^9^, ^10^ Notably, the inactivation of the cones also reduces light avoidance,^8^ and recent work indicates that the S‐cones and M‐cones provide opposing inputs to the ipRGCs that modulate light‐aversive behavior.^11^

These photoreceptor signals have been shown to mediate aversive behavior, at least in part through the somatosensory trigeminal system. In rats, exposure to very bright light produces neural activation within the trigeminal nucleus caudalis, the subnucleus associated with pain transmission.^12^ Responses to bright light are found in the posterior somatosensory thalamus in neurons that also receive dural afferents.^13^ Optogenetic stimulation of this thalamic nucleus evokes light‐aversive behavior in mice.^14^

The interaction of light and trigeminal signals in the perception of visual discomfort is a feature of migraine headaches.^15^, ^16^, ^17^ Signals from the ipRGCs appear to be amplified in individuals with migraine,^18^, ^19^ who commonly experience increased light sensitivity during and sometimes even between attacks.^20^, ^21^, ^22^, ^23^, ^24^, ^25^, ^26^, ^27^, ^28^ A key preclinical model of migraine is the induction of light aversive behavior in mice by administration of calcitonin gene‐related peptide (CGRP).^29^, ^30^, ^31^, ^32^ This neuropeptide modulates nociceptive pathways, mediates neurogenic inflammation, and is a highly efficacious therapeutic target in migraine.^33^, ^34^ An important, unanswered question is whether the effect of CGRP is specifically to enhance aversive behavior evoked by ipRGC signals.

Here, we sought to replicate prior rodent studies by establishing the photoreceptor basis of light‐aversive behavior and then extend this paradigm to examine whether CGRP administration amplifies ipRGC signals for light avoidance. We modified a previously described light aversion behavioral assay^29^, ^30^, ^31^, ^32^ to deliver spectral modulations of light that target specific photoreceptors using the technique of silent substitution.^35^ In these studies, the use of a transgenic mouse, in which the mouse M‐cone is replaced with the human L‐cone, avoided spectral overlap between Mel and the mouse M‐cone, allowing our light modulations to produce a larger differential targeting of the photoreceptors.^36^ We specifically examined aversion to Mel and cone stimulation, both in isolation and in combination, and explored if S‐cone stimulation could oppose the aversive effects of Mel and L‐cone stimulation. We used a genetic ablation of Mel‐containing RGCs to determine if our behavioral observations depended on ipRGC function. Finally, we examined whether repeated exposure to CGRP enhanced light avoidance by the ipRGCs.

## METHODS

### Animals

Wild‐type (WT) and transgenic strains of mice used for experiments included: (1) WT C57BL/6J mice (Jackson Labs, Bar Harbor, ME); (2) transgenic human L‐cone cone knock‐in mice (HLCKI, B6.129‐Opn1mw^tm1(OPN1LW)Nat^/J; Jackson Labs, Bar Harbor, ME),^37^ and (3) adult‐onset genetic ablation of ipRGCs (Opn4^aDTA/aDTA^, *Opn4* ^*tm4.1(DTA)Saha*^ */J*),^38^ which were crossed with HLCKI mice. Note that ablation of ipRGCs is thought predominantly due to M1 loss, as M1 ipRGCs have the highest level of Mel (Opn4) expression, facilitating apoptosis by diphtheria. In contrast, M2‐M6 ipRGCs have much lower levels of Mel expression and thus produce lower levels of diphtheria, so are less likely to undergo apoptosis.^4^, ^39^ C57BL/6J mice were shipped at 9 weeks of age and then acclimated in our animal facilities for a minimum of 7 days prior to testing. Alternatively, C57BL/6J mice (Jackson Labs, Bar Harbor, ME) were bred, and subsequent litters were also used for testing.

Male and female mice were tested between 10 and 20 weeks of age, except for experiments involving HLCKI x Opn4^aDTA^ mice, which were tested between 21 and 34 weeks of age. In general, ~10–20 mice per treatment condition were tested, which was based on group sizes and variability in light avoidance behavior observed in a similar prior study.^32^ In a few cases, larger group sizes were reported when two separate cohorts were tested to confirm the validity of findings. All animals were housed in groups of 2–5 per cage in standard conditions, on a 12‐h light cycle (on at 0700 ET, off at 1900 ET), and with access to water and food ad libitum. Animal care procedures were approved by the University of Pennsylvania Animal Care and Use Committee and performed in accordance with the standards set by the National Institutes of Health.

### Intraperitoneal drug administration

CGRP was diluted with Dulbecco PBS (Hyclone) as the vehicle (Veh). Mice were injected with either 0.1 mg/kg of rat α‐CGRP (Sigma) or Veh, which was administered at 10 μL/g body weight with BD ultrafine 31 g insulin syringes. Injections were performed by E.A.K. or A.C. just prior to behavioral testing. Animals were gently restrained but not anesthetized during injection.

### Light aversion testing chamber

The open field and infrared tracking equipment (Med Associates Inc., St. Albans, VT) have been previously described.^31^ The field was divided into two equal‐sized zones with an opening for free movement between the two zones. Each zone contained a panel of light‐emitting diodes (LEDs) with three different, narrow‐bandwidth LEDs with peak wavelength intensities of: 365 nm (realUV LED Strip Lights, Waveform Lighting), 460 nm (SimpleColor Blue LED Strip Lights, Waveform Lighting), and 630 nm (SimpleColor Red LED Strip Lights, Waveform Lighting). Each set of LEDs was digitally controlled. See Supplemental Materials for additional details.

### Non‐targeted light aversion behavior assay

On the day of the experiment, mice were briefly transported to the testing room and then allowed to acclimate within their home cage to the testing room for at least 1 h with standard overhead fluorescent lighting. All sound‐generating equipment was turned on during acclimation and remained on until testing was complete. Behavioral testing was performed between 0900 and 1830 local time. See Supplemental Materials for a description of ambient light conditions.

Following acclimation, mice were placed into the front zone of the cage and allowed to explore both zones freely for 60 min. From 0 to 30 min, both LED panels were off, and then, from 30 to 60 min, one of the panels was randomly turned on. Here, the LEDs in the light zone were set to a specific contrast level, which we defined as a proportion of the maximal contrast level ranging from 1.00 (2440 photopic lux; 8624 melanopic lux) to 0.05 (116 photopic lux; 388 photopic lux). The opposing LED panel remained off in a standard light–dark box paradigm. A plexiglass panel beneath the LEDs was used to diffuse light; we later discovered that this panel fully blocked UV light. Thus, there was essentially no UV light transmission for this set of experiments.

Animals were tested ~48 h later following the same protocol; however, mice were administered CGRP or Veh as described previously and then immediately placed back in the chamber for 60 min under the same light paradigm described above.

### Photoreceptor‐targeted light aversion behavior assay

As described for the non‐targeted assay, following acclimation to the room, mice were placed into one of the zones and allowed to explore both zones freely for 60 min. From 0 to 30 min, both LED panels were turned on with the UV, blue, and red LEDs, each set to 50% of max setting. For 30 to 60 min, the LED panels illuminating both zones were adjusted to create a selective difference in contrast upon the targeted photoreceptor(s) using the principles of silent substitution.^35^ To accomplish this, the intensities of the UV, blue, and red LEDs were digitally scaled in each LED panel such that the isomerization rate of non‐targeted photoreceptors was kept constant between the two zones, thus “silent,” while the spectral content of the LEDs for the targeted photoreceptor was scaled to create greater stimulation in one zone compared to the other zone. This approach avoided the potential complication of spectral overlap between the targeted and non‐targeted photoreceptors and produced an absolute contrast on a targeted class of photoreceptor (e.g. 59.88% on Mel), which is the Weber contrast created by the difference between the background of the high and low contrast zones. For each targeted photoreceptor, we presented relative contrast levels that varied from 1.00, 0.75, and 0.50. These are a proportion of the maximum targeted photoreceptor modulation presented (e.g., 59.88%, 44.91%, 29.94%, respectively, absolute contrast was achieved on Mel; see Table 1 for the absolute percent contrast for each photoreceptor under each targeted photoreceptor modulation for 1.00 relative contrast). The targeted photoreceptor classes included mouse S‐cone, Mel, and/or human L‐cone (see Figure 1E–G for spectral sensitivity functions). Light flux describes the stimulus that targets all three photoreceptors equally. The UV‐blocking diffusion panel was not used for this set of experiments.

**TABLE 1:** Absolute percent contrast levels for each photoreceptor type generated by the spectral modulations used for the photoreceptor‐targeted light aversion behavior assay.

Photoreceptor direction	Mouse S‐cone	Mouse Mel	Mouse rod	Human L‐cone
S‐cone	99.61	0.00	0.80	0.00
Mel	0.00	59.88	58.97	0.00
L‐Cone	0.00	0.00	0.26	35.11
Light flux	100.00	100.00	100.00	100.00

Abbreviation: Mel, melanopsin.

### Chronic intermittent administration of CGRP

Modifying a previously described protocol,^40^ animals were administered either intraperitoneal (ip) CGRP or Veh as described above every‐other‐day over 9 days (i.e., days 1, 3, 5, 7, 9). On days 1, 5, and 9, animals were placed in the behavior chamber immediately after administration of CGRP or Veh and tested as per the photoreceptor‐targeted light aversion behavioral assay. On days 3 and 7, animals were returned to their home cages following ip injections.

### Statistical Analysis

Data were processed and analyzed using Microsoft Excel (Microsoft Corporation), RStudio (version 2024.090 + 375, Posit‐ Software, PBC), and Prism 10 for Mac OS X (Graphpad Software, Inc.). Results were reported as mean ± standard error of the mean. Time in the light zone or the high contrast zone for the targeted photoreceptor was reported as 5‐min binned intervals over the 60‐min testing duration (12 intervals in total). For each interval, time in the light zone (or the high contrast zone) was normalized to the mean time spent in that zone over the first six intervals (0 to 30 min). Asymptote value (AV) was defined as 1 minus the mean normalized time in the light zone (or the high contrast zone) over the final 3–5 min intervals (45 to 60 min).

A two‐way analysis of variance (ANOVA) was used to assess the effect of a specific condition (e.g., specific photoreceptor(s) targeted, genotype) over the 60‐min testing duration. This test was conducted for the normalized time in the light (or high contrast) zone, and subsequent post‐hoc analyses were performed to compare differences at a specific 5‐min interval using the Sidak multiple comparisons test. A Mann–Whitney test was performed when comparing the mean asymptotic value between two different conditions.

Mice were excluded from the analysis due to equipment or software failure. Mice were also excluded for atypical exploratory behavior based on one of two criteria: (1) failing to explore both zones during one of the 5‐min intervals during the 0 to 30 min acclimation period (*n* = 6 for all experiments) or being identified as an outlier using the ROUT method (Q = 1%; *n* = 8 for all experiments).

## RESULTS

### Light avoidance behavior depends on the contrast level

First, we determined how the degree of light avoidance in a light–dark box changes as the contrast level between the dark zone and the light zone increases. In our non‐targeted light avoidance behavioral assay, WT (C57BL6/J) mice were observed for 30 min while mice acclimated to the box with LEDs off in both zones. During the testing period, one zone was illuminated by narrow‐bandwidth LEDs with peak wavelength intensities at 460 nm (blue) and 630 nm (red); a third LED class (UV, 365 nm) was present but filtered by a UV‐blocking panel (Figure 1B). The contrast of the light zone was manipulated by increasing the intensity of the LEDs from their maximal setting, 0.05, 0.15, 0.25, 0.50, and 1.00. Under these testing conditions, mice were observed for an additional 30 min. Light avoidance was assessed by measuring the time in the light zone, which was normalized to the mean time mice spent in that zone during a 30‐min acclimation period.

With the initiation of 1.00 contrast after the 30‐min interval, mice (*n* = 24) spent progressively less time in the illuminated zone, tending to reach an asymptotic level of avoidance after 45 min. We summarized light avoidance behavior using an asymptotic value (AV; 1 minus the mean, normalized time spent in the illuminated zone in the final 15 min of the assay). AV >0 reflects light avoidance, and AV <0 reflects light preference. For 1.00 contrast, the AV was 0.67 ± 0.06.

**FIGURE 2:**
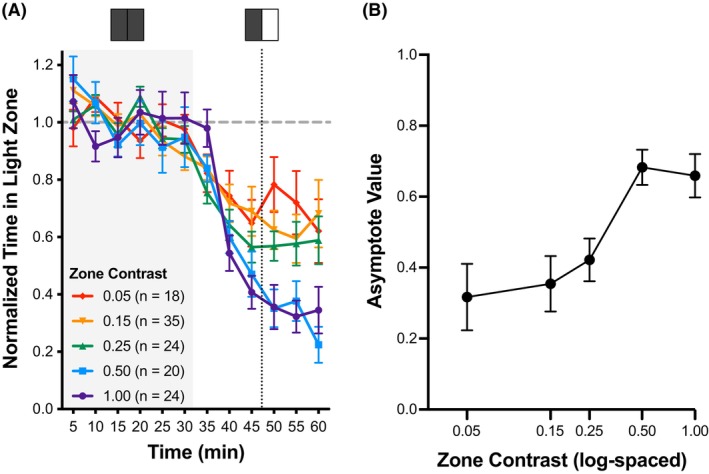
Contrast dependence of light avoidance behavior. (A) Normalized time spent in the light zone. The zone contrasts tested were: (1) 0.05 (red); (2) 0.15 (orange); (3) 0.25 (green); (4) 0.50 (blue); and (5) 1.00 (purple). Wild‐type C57BL6/J mice were tested under non‐targeted conditions. (B) Using the asymptote value (AV), light avoidance behavior is expressed as a function of log‐spaced zone contrast. The AV is calculated from 1 minus the mean normalized time in light during the 50‐, 55‐, and 60‐min intervals. Error bars indicate mean ± SEM. [Color figure can be viewed at wileyonlinelibrary.com]

When contrast of less than 1.00 was used, the chamber preference evolved more slowly and with less asymptotic avoidance (Figure 2A). We examined AV as a function of contrast and observed a clear contrast‐dependent response (Figure 2B). The dependence of avoidance behavior upon contrast follows a sigmoidal function and has apparent threshold and ceiling effects.

### Mel and L‐cone stimulation elicit light avoidance

We modified the light avoidance behavioral assay to target specific photoreceptor classes (Figure 1C). From 0 to 30 min, mice were acclimated to the box with both zones set to a background light level of 50%, which is half‐maximum of all three LED classes. During the testing period (30–60 min), the intensities of the UV, blue, and red LEDs were individually digitally adjusted to produce spectral profiles that provided targeted, differential photoreceptor contrast between the two zones (Figure 1E–G). To better isolate Mel versus cone function, we tested transgenic mice with a red cone knock‐in (HLCKI, B6.129‐Opn1mw^tm1(OPN1LW)Nat^/J), replacing the mouse M‐cone and, thus, shifting the spectral sensitivity of that cone into the red range of the visual spectrum (Figure 1D).

**FIGURE 3:**
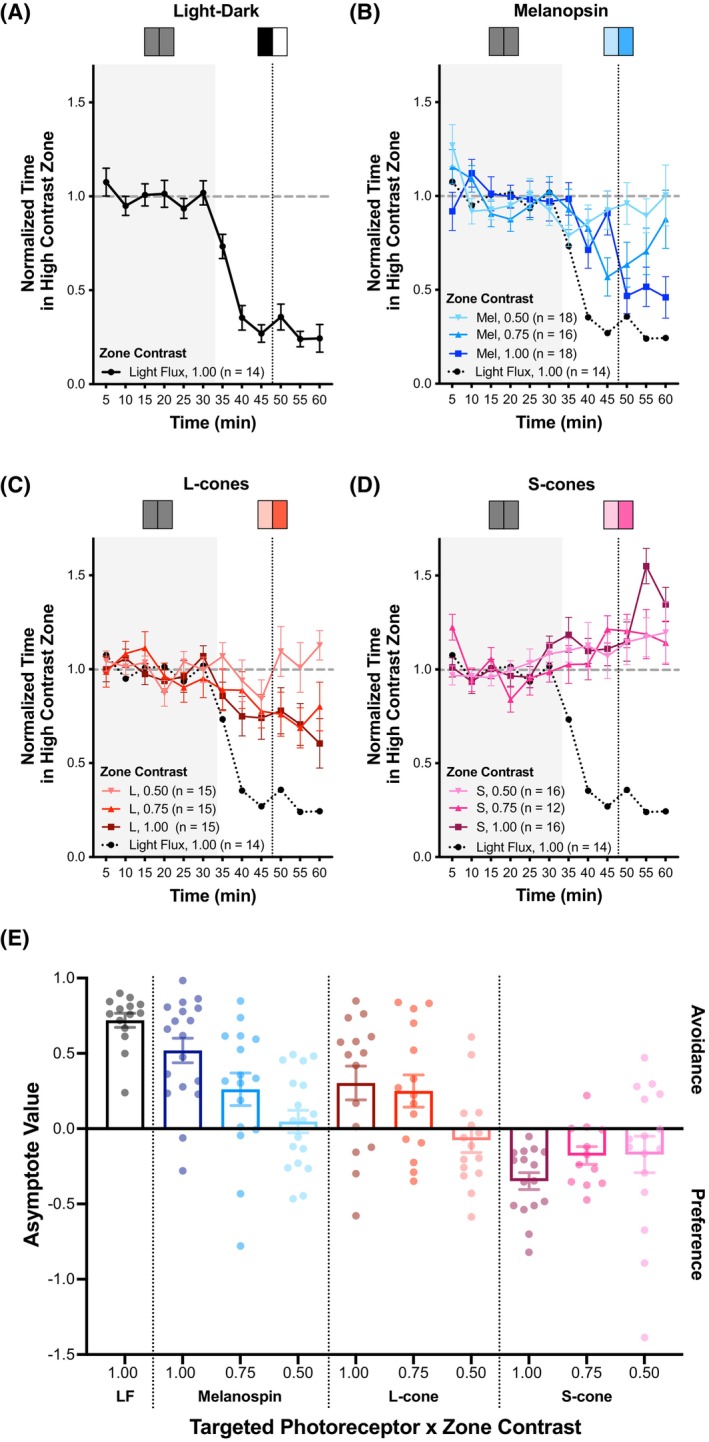
Effect of targeted photoreceptor stimulation on light avoidance or preference behavior. For panels A‐D: Normalized time spent in the high contrast zone. The targeted photoreceptors included: (A) light flux, (B) melanopsin (Mel), (C) human L‐cone, and (D) mouse S‐cone. HLCKI mice were tested under targeted photoreceptor conditions. Relative contrast levels between zones were 0.50, 0.75, and 1.00. (B–D) The effect of light flux (dotted line) is included as a reference. (E) Asymptote value (AV) for each photoreceptor targeted across each zone contrast. Targeted photoreceptors tested include: (1) light flux (black); (2) Mel: 0.50 (light blue), 0.75 (medium blue), 1.00 (dark blue); (3) human L‐cone: 0.50 (light red), 0.75 (medium red), 1.00 (dark red); (4) mouse S‐cone: 0.50 (light purple), 0.75 (medium purple), 1.00 (dark purple). AV >0 reflects light avoidance, and AV <0 reflects light preference. Plot symbols correspond to individual animals; bars indicate mean ± SEM. [Color figure can be viewed at wileyonlinelibrary.com]

We first confirmed that a full light/dark manipulation would produce light avoidance using this modified procedure. After acclimation to the two, half‐illuminated zones, the lighting was adjusted to produce a maximal absolute contrast on all three photoreceptors equally, creating 1.00 relative contrast between the two zones (termed “light flux”). As seen in our initial measurement (Figure 2), HLCKI mice showed robust light avoidance to light flux (Figure 3A,E).

We next measured light avoidance elicited by differential spectral contrast that targeted specific photoreceptors. Targeted stimulation of Mel led to light avoidance, and the degree of avoidance was contrast‐dependent (Figure 3B,E). Avoidance was also evoked by targeted L‐cone stimulation, and again, a contrast‐dependent effect was seen (Figure 3C,E). For both Mel and L‐cone stimulation, 50% stimulus contrast was below the threshold required to produce a measurable avoidance response.

### S‐cone stimulation elicits light preference

A prior study reported that, when combined with L‐cone stimulation, concurrent S‐cone stimulation reduces avoidance behavior;^11^ this effect is believed to result from inhibitory S‐cone extrinsic input on M1 ipRGCs.^41^ We therefore examined the behavioral responses to isolated S‐cone stimulation. Here, we observed that HLCKI mice spent more time in the high‐contrast zone with S‐cone stimulation, which tended to increase over the testing period (Figure 3D). This light preference behavior was contrast‐dependent (Figure 3E).

### S‐cone stimulation opposes Mel and L‐cone light avoidance

**FIGURE 4:**
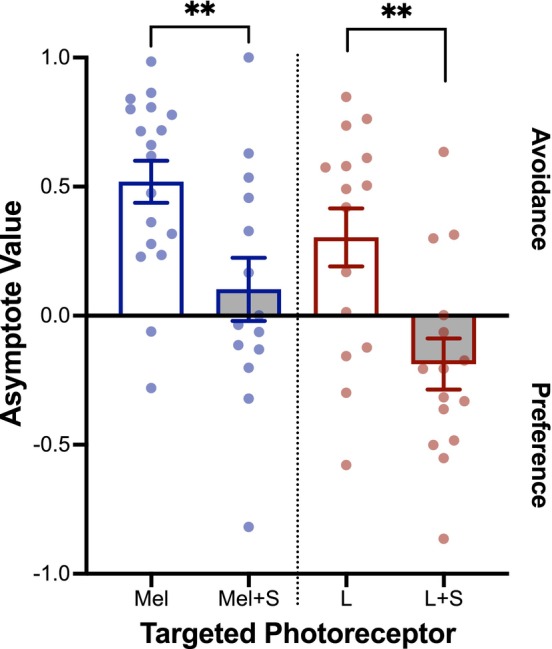
Effect of combining S‐cone stimulation to melanopsin (Mel) or human L‐cone stimulation on light avoidance or preference behavior. Asymptote value (AV) for each photoreceptor targeted across each zone contrast. Targeted photoreceptors tested include: (1) Mel alone (blue, empty bar), *n* = 18; (2) Mel and mouse S‐cone (blue, gray bar), *n* = 14; (3) human L‐cone alone (red, empty bar), *n* = 15; (4) human L‐cone and mouse S‐cone (red, gray bar), *n* = 15. AV >0 reflects light avoidance, and AV <0 reflects light preference. Plot symbols correspond to individual animals; bars indicate mean ± SEM. **p < 0.01. [Color figure can be viewed at wileyonlinelibrary.com]

To examine this inhibitory effect further, we examined whether adding S‐cone stimulation could reduce the light avoidance to Mel or L‐cone stimulation. Combining equal absolute S‐cone contrast with Mel contrast led to minimal light avoidance in HLCKI mice, and significantly less avoidance was found from Mel stimulation alone (U = 58; *p* = 0.009; *n* (Mel) = 18; *n* (Mel+S) = 14) (Figures 4, S1A). Similarly, adding equal absolute S‐cone contrast to L‐cone contrast overcame avoidance of L‐cone stimulation and led to a small light preference, which significantly differed from L‐cone stimulation alone (U = 44; *p* = 0.004; *n* (L) = 15; *n* (L + S) = 15) (Figure 4, S1B).

These findings are consistent with an S‐cone inhibitory input on ipRGCs that opposes the excitatory, intrinsic signals from Mel and the excitatory, extrinsic input from L‐cones.

### ipRGCs are key mediators of light avoidance/preference behavior

**FIGURE 5:**
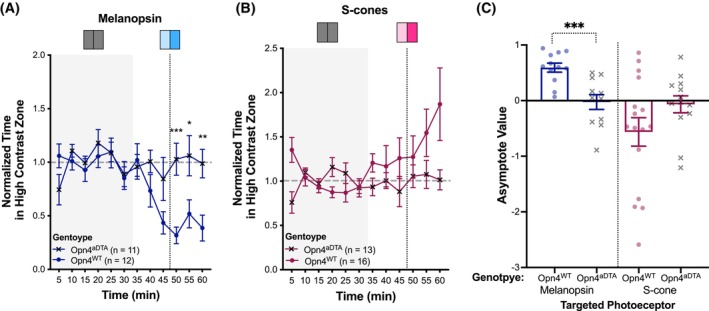
Effect of ablating melanopsin (Mel)‐containing RGCs (ipRGCs) on light avoidance to Mel stimulation or preference to mouse S‐cone stimulation. (A,B) Normalized time spent in the high contrast zone. The targeted photoreceptors included: (A) Mel (dark blue) and (B) mouse S‐cone (dark purple). HLCKI x Opn4^aDTA^ mice (n [Mel] = 11; *n* [mouse S‐cone] = 13, x symbol) with adult‐onset ablation of ipRGCs and control mice, HLCKI x Opn4^WT^ (n (Mel) = 12; *n* (mouse S‐cone) = 16, closed circle), were tested under targeted photoreceptor conditions. The relative contrast level between zones was 1.00. (C) Asymptote value (AV) for each photoreceptor and genotype as outlined in panels A and B. Each dot represents an individual animal. AV >0 reflects light avoidance, and AV <0 reflects light preference. Plot symbols correspond to individual animals; bars indicate mean ± SEM. *p = 0.026; **p = 0.008; ***p < 0.001. [Color figure can be viewed at wileyonlinelibrary.com]

To assess whether ipRGCs are responsible for mediating light avoidance/preference behavior, we observed these behaviors in HLCKI mice crossed with transgenic mice that have adult‐onset genetic ablation of Mel‐expressing ipRGCs (Opn4^aDTA/aDTA^ or Opn4^aDTA^) and compared their responses to mice with the WT Mel gene (Opn4^+/+^ or Opn4^WT^). To ensure ablation of ipRGC was complete prior to testing, mice were examined after 20 weeks of age. In response to Mel‐directed stimulation, HLCKI x Opn4^aDTA^ mice showed no light avoidance, while HLCKI x Opn4^WT^ mice showed robust aversion to Mel stimulation (Figure 5A,C). This effect on AV was significantly different (U = 12; *p* < 0.001; *n* (Opn4^aDTA^) = 12; *n* (Opn4^WT^) = 11) (Figure 5A,C). Furthermore, when examining the behavior over the 60‐min period, genotype had a significant effect (F (1,21) = 9.16, *p* = 0.006). In response to S‐cone stimulation, HLCKI x Opn4^aDTA^ mice showed essentially no light preference as compared to HLCKI x Opn4^WT^ mice (Figure 5B,C); however, this reduced effect did not reach statistical significance (U = 60; *p* = 0.132; *n* (Opn4^aDTA^) = 13; *n* (Opn4^WT^) = 16). Similarly, when examining the behavior over the 60‐min period, the genotype effect did not reach significance (F (1,27) = 3.11, *p* = 0.089). Note that HLCKI x Opn4^WT^ did not show avoidance of L‐cone stimulation, but rather demonstrated light preference (Figure S2), contradicting our prior observations in younger (10–20 weeks) HLCKI mice (Figure 3C,E). Nevertheless, this light preference was nearly eliminated in HLCKI x Opn4^aDTA^ mice. However, this genotype effect did not reach statistical significance (F (1,32) = 1.97, *p* = 0.17).

Overall, these findings suggest that ipRGCs are responsible for transmitting light signals to subcortical and cortical circuits, thereby producing light avoidance/preference behavior.

### CGRP enhances Mel‐mediated light avoidance

Mason and colleagues found that WT mice show greater light avoidance following a single administration of ip CGRP as compared to Veh;^32^ however, it required very bright white light to induce the behavior. Here, we examined whether CGRP could increase light avoidance in the non‐targeted light avoidance behavior assay. We tested C57BL6/J mice 2 days after their initial testing, as described in Figure 2. We administered either ip CGRP (0.1 mg/kg) or ip Veh to WT mice just before the acclimation period (Figure S2A,B). CGRP‐treated animals tended to have greater light avoidance at 0.15, 0.50, and 1.00 contrast than Veh animals; however, these effects were non‐significant. While CGRP appeared to enhance light avoidance at higher contrast levels, negative valence responses (e.g., fear) to ip injections may have limited our ability to detect group differences between CGRP and Veh‐treated mice. Repeated testing, allowing for acclimation to the chamber, has previously reduced this Veh‐effect.^31^, ^32^

**FIGURE 6:**
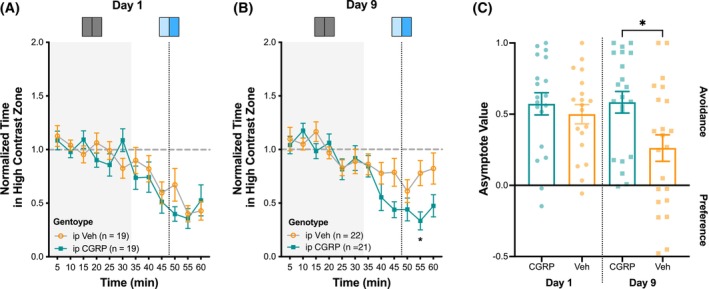
Effect of chronic intermittent administration of calcitonin gene‐related peptide (CGRP) on light avoidance behavior to melanopsin (Mel) stimulation. (A, B) Normalized time spent in the high contrast zone with the Mel as the targeted photoreceptor at a relative contrast level of 0.75. Human L‐cone knock‐in (HLCKI) mice were administered ip CGRP (0.1 mg/kg) or vehicle (Veh) on days 1, 3, 5, 7, and 9, with testing conducted on (A) day 1, day 5 (see Figure S3), and (B) day 9. Error bars indicate ±SEM. (C) Asymptote value (AV) for each photoreceptor and genotype as outlined in panels A and B. The groups tested included ip Veh and ip CGRP. Each dot represents an individual animal. AV >0 reflects light avoidance, and AV <0 reflects light preference. Plot symbols correspond to individual animals; bars indicate mean ± SEM. *p < 0.05. [Color figure can be viewed at wileyonlinelibrary.com]

Next, we examined whether chronic intermittent administration of CGRP could enhance the light avoidance response to Mel stimulation. Here, HLCKI mice were administered either ip CGRP (0.1 mg/kg) or Veh every other day over a nine‐day period. Following treatment on days 1, 5, and 9, light avoidance of Mel at 0.75 contrast was examined. This moderate level of contrast was selected to determine whether chronic intermittent administration of CGRP could further increase the moderate level of avoidance observed in Figure 3B,E. On day 1, light avoidance of Mel was similar between CGRP (AV: 0.57 ± 0.8) and Veh‐treated (AV: 0.50 ± 0.07) mice (Figure 6A,C). In Veh‐treated mice, light avoidance decreased on day 5 (AV: 0.30 ± 0.09) (Figure S3) and remained low on day 9 (AV: 0.26 ± 0.09). This observation aligns with previous findings that mice acclimate to the light avoidance behavioral assay with repeated testing.^31^, ^32^ In CGRP‐treated mice, light avoidance also decreased on day 5 (AV: 0.28 ± 0.11) but increased on day 9 (AV: 0.58 ± 0.08). When examining the behavior over the 60‐min testing period on day 9, treatment had a significant effect (*F* (1, 41) = 5.70, *p* = 0.022) with CGRP‐treated animals spending less time in the high Mel contrast zone than Veh‐treated animals starting at the 40‐min interval. Per analysis of the AV on day 9, CGRP‐treated mice showed significantly greater light avoidance of Mel than Veh‐treated mice (U = 136; *p* = 0.020; *n* (CGRP) = 21; *n* (Veh) = 21) (Figure 6C). This suggests that chronic intermittent administration of CGRP can enhance light avoidance of Mel stimulation.

## DISCUSSION

Our study provides novel evidence of photoreceptor‐specific light avoidance responses in a pre‐clinical migraine model. We find that light avoidance behavior in the mouse is sensitive to relatively small differences in light contrast, following a lawful stimulus–response relationship. Similar to prior human work,^11^ the pattern of response to different photoreceptor combinations implicates the ipRGCs as the source of the light avoidance signal. Further support for this proposition was found in the effect of ablating ipRGCs upon light avoidance and preference. A significant additional contribution of this study was our demonstration that chronic intermittent administration of CGRP amplifies the effect of these photoreceptor manipulations upon light avoidance behavior.

As previously reported,^5^, ^6^, ^11^ we found that mice avoid the testing chamber containing a light spectrum enriched for Mel and L‐cone stimulation. In contrast, increasing S‐cone stimulation induced a zone preference, aligning with a prior observation.^11^ The opposite effects of L‐ and S‐cone stimulation upon behavior are consistent with the opponent synaptic inputs derived from these cone classes upon the M1 class of ipRGCs.^41^ We also found that adult‐onset genetic ablation of ipRGCs eliminated avoidance of Mel stimulation and preference for S‐cone stimulation. This ablation procedure is thought to principally inactivate the M1 ipRGC class.^4^, ^38^ Taken together, our results suggest that the M1 class is both a necessary and sufficient signal for maximal light avoidance, which can be downregulated by S‐cone input.

Our study employed silent substitution approaches to target photoreceptor classes. As discussed below, this approach has some limits in the strength of the photoreceptor stimulus that may be produced. A distinct advantage, however, is that silent substitution stimuli may also be used to study the intact human visual system, thereby increasing the translational relevance of this rodent work. Indeed, our results may be related to prior studies in humans that have shown that selective Mel and cone stimulation produce visual discomfort in a manner suggestive of the integration of these signals in the ipRGCs.^18^ This prior clinical study did not examine isolated S‐cone stimulation; therefore, it remains unknown whether this manipulation can be used to decrease visual discomfort in humans. Support for this idea, however, is found in studies of the human pupil response (which is also mediated by M1 ipRGC signals)^4^ in which isolated S‐cone stimulation produces a paradoxical, opponent dilation of the pupil.^42^ Moreover, FL‐41 lenses, which have been shown to reduce photophobia,^43^ may be effective not only because they reduce the transmission of wavelengths of light that stimulate Mel, but also allow for greater transmission of light that can stimulate S‐cones relative to Mel and the L‐ and M‐cones.

An important contribution of our work is demonstrating the photoreceptor basis of light avoidance following CGRP administration. Both peripheral and central CGRP administration can induce light aversive behavior, likely by independent mechanisms.^29^, ^30^, ^31^, ^32^ In our non‐targeted assay, we found that CGRP treatment produced greater light avoidance than Veh‐treated mice, but only at high illuminance. Light avoidance following Veh administration in our non‐targeted assay appeared to be greater than that reported by Mason and colleagues; this difference may reflect variations in experimental procedure (e.g., mice in the current study were not acclimated to the chamber twice prior to receiving CGRP or Veh). Ours is the first to report that chronic intermittent administration of CGRP leads to increased light avoidance. CGRP levels are elevated during a migraine attack;^33^ thus, chronic intermittent administration of CGRP may model the effect of tonically elevated CGRP in high‐frequency migraine states. More frequent migraine attacks appear to be associated with interictal photophobia; one study observed lower light thresholds in those with chronic migraine as compared to episodic migraine and healthy controls.^44^ Here, the heightened light avoidance behavior was in response to moderate Mel stimulation, suggesting that CGRP potentiates aversive ipRGC signals. Future studies should validate this response in WT mice and determine whether S‐cone stimulation or ipRGC ablation can attenuate the enhanced light avoidance in response to chronic intermittent CGRP administration.

CGRP‐induced potentiation of ipRGCs likely stems from CGRP repeatedly activating peripheral trigeminal afferents. Chronic intermittent administration of CGRP led to periorbital allodynia in mice by day 5.^40^ As we did not observe an effect until day 9, modulation of ipRGC signals may require greater central sensitization and is perhaps mediated by second‐order trigeminal neurons in the trigeminal nucleus caudalis and other subcortical circuits. We also note that extending the duration of chronic intermittent administration of CGRP could produce even greater amplification of aversive ipRGC signals and potentially facilitate light avoidance at subthreshold levels of contrast, which would be indicative of a primed state. We hypothesize that chronic intermittent administration of CGRP induces a cascade of neuroplastic changes in subcortical pathways, including the upregulation of glutamatergic signaling, activation of satellite glia, and synaptic modifications of interneurons. Understanding the neural network that mediates photophobia and how it becomes sensitized in migraine is a ripe area for future investigation.

The magnitude of the light avoidance effects we observed was smaller than has been reported in other studies.^29^, ^30^, ^31^, ^32^ Our use of photoreceptor targeting is a likely explanation. Non‐selective increases in broadband light from darkness can produce unbounded increases in photoreceptor contrast. Such a stimulus drives all intact photoreceptor classes simultaneously (and is arguably more reflective of ecological contexts) but does not allow for inferences regarding differential contributions from photoreceptors. Here, we accepted the stimulus intensity limitations of the silent substitution approach, given its desirable inferential and translational properties.

Unexpectedly, we were unable to replicate the avoidance of L‐cone stimulation in HLCKI mice, as shown in Figure S3, which we had initially observed in Figure 3C,E. We cannot account for this discrepancy in results. One hypothesis is that mice prefer the high contrast L‐cone chamber due to a small elevation in temperature from the red LEDs. This may particularly be the case when the room temperature is lower due to air conditioning. Until this source of variation in response can be identified, the L‐cone avoidance results should be treated with caution.

## CONCLUSIONS

ipRGCs play a key role in the perception of light as aversive in humans and mice, integrating the excitatory input from intrinsic Mel stimulation and extrinsic L‐cone input with inhibitory extrinsic input from S cones. Mice appear to be sufficient models of light sensitivity, allowing for future dissection of the downstream neural circuitry in which ipRGC signals interact with trigeminal pathways and negative valence networks. CGRP‐mediated central sensitization of the trigeminal system is likely one of multiple neural processes that can modulate ipRGC signals, leading to increased light sensitivity.

## AUTHOR CONTRIBUTIONS

**Eric A. Kaiser:** Conceptualization; investigation; funding acquisition; writing – original draft; methodology; validation; visualization; writing – review and editing; formal analysis; data curation. **Audrey Cavanah:** Investigation; writing – review and editing. **Geoffrey K. Aguirre:** Conceptualization; funding acquisition; writing – review and editing; writing – original draft; methodology; visualization; software; supervision; resources; data curation. **Frances E. Jensen:** Supervision; resources; project administration; funding acquisition; writing – review and editing; methodology; conceptualization.

## FUNDING INFORMATION

National Institute of Neurological Disorders and Stroke, Grant Award Number: K08 NS120595; National Eye Institute, Grant Award Number: P30 EY001583; Amgen Competitive Grant Program in Migraine Research.

## CONFLICT OF INTEREST STATEMENT

**Eric A. Kaiser** has received royalties (Lundbeck, formerly Alder Biopharmaceuticals) from patents involving the use of anti‐CGRP monoclonal antibodies to treat photophobia. **Audrey Cavanah, Geoffrey K. Aguirre**, and **Frances E. Jensen** have no financial disclosures.

## Supporting information


Figure S1.
