# Supplementary material for: Amplifying and ameliorating light avoidance in mice with photoreceptor targeting and calcitonin gene‐related peptide sensitization
Source: Headache. 2025 Dec 15;66(1):132–43. doi: 10.1111/head.70018 (PMC12849529; doi:10.1111/head.70018)
Supplement: Supplementary file 1 — Figure S1. [file HEAD-66-132-s001.docx]

**Supplemental Materials:**

**S1. Methods:**

*S1.1 Ambient Lighting Conditions*

With lights on in the housing room, the ambient lighting was 780 photopic lux and 320 melanopic lux, but this ranged from 0.812 to 516 photopic lux and 0.042 to 182 melanopic lux within a cage, depending on the position on the rack. For the room in which behavior was assessed, ambient light levels were 451 photopic lux and 222 melanopic lux, but light levels decreased to 57 to 155 photopic lux and 22 to 66 melanopic lux within cages during acclimation.

*S1.2 Light aversion testing chamber – additional details*

Behavior was assessed in a plexiglass open field (27.0 cm wide x 27.0 cm deep x 20.3 cm high) containing two sets of 16-beam infrared arrays (Med Associates Inc., St. Albans, VT). The field was divided into two equal-sized zones by a custom-made acrylic insert that was opaque to visual light but transmitted infrared light (ePlastics, San Diego, CA). The insert was a five-sided, opaque, dark-colored box with a top but no floor. In the center, a removable divider split the chamber into two equal zones and contained an opening (5.2 cm x 6.8 cm), allowing free movement between zones. Each zone contained two vents, one of which contained a 25 mm x 10 mm, 5 V fan to allow unrestricted airflow and reduce the heat generated by the LED panels. A baffle covered the vents to reduce light penetration into the box. Finally, the roof of the box contained a panel of LEDs over each zone. Each testing chamber was enclosed in a sound-attenuating cubicle (56 cm wide x 38 cm deep x 36 cm high), which contained a fan for ventilation (Med Associates Inc.).

Each LED panel was connected to a control box that provided power to the lights, as well as a 32-bit ARM core microcontroller (Arduino Due, Arduino). This allowed for independent, 12-bit digital control of the intensity of the UV, blue, and red LEDs in each panel. Custom MATLAB (The MathWorks, Inc, Natick Massachusetts) code was used to set and change contrast levels between the zones.

We performed a calibration of the LED panel using a radiometer (SpectraScan Spectroadiometer PR-670, JADAK, North Syracuse, NY), which measured the spectral emissions of the blue and red LEDs and confirmed a linear gamma function. This photometer could not directly measure UV light, so we relied on tabular values provided by the LED manufacturer to extrapolate a linear gamma function based on our findings from the red and blue LEDs.

Infrared tracking of mouse movement was recorded and analyzed by using Activity Monitor v6.02 (Med Associated Inc.) software.

We measured a 2° C difference in temperature between the chambers under dark and 1.00 contrast conditions. Prior work has indicated that this temperature difference is unlikely to change rodent chamber preference.^45^

**Supplemental Figures**

**Figure S1. Effect of combining S-cone stimulation to melanopsin or human L-cone stimulation on light avoidance or preference behavior.** For panels A-B: Normalized time spent in the high contrast zone. The targeted photoreceptors included: **A**. melanopsin ± S-cone **B.** human L-cone ± S-cone. HLCKI mice were tested under targeted photoreceptor conditions including: 1) melanopsin alone (dotted dark blue line, closed circle), n = 18; 2) melanopsin and mouse S-cone (gray line, open circle), n = 14; 3) human L-cone alone (dotted red line, closed circle), n = 15; 4) human L-cone and mouse S-cone (gray line, open circle), n = 15. The relative contrast level between zones was 1.00. Error bars indicate mean ± SEM. *p < 0.05. ***p < 0.001.

**Figure S2. Effect of ablating melanopsin-containing RGCs (ipRGCs) on light avoidance to human L-cone stimulation.** Normalized time spent in the high contrast zone. The targeted photoreceptor was human L-cone (dark red). HLCKI x Opn4^aDTA^ mice (n = 13, x symbol) with adult-onset ablation of ipRGCs and control mice and HLCKI x Opn4^WT^ (n = 21, closed circle) were tested under targeted photoreceptor conditions. The relative contrast level between zones was 1.00. Error bars indicate mean ± SEM.

**Figure S3. Effect of a single administration of CGRP on light avoidance behavior to non-targeted light stimulation.** In panel A-B, normalized time spent in the light zone. Contrast tested were: 1) 0.05 (red), n = 9; 2) 0.15 (orange), n(Veh) = 14, n(CGRP) = 16; 3) 0.25 (green), n = 12; 4) 0.50 (blue), n = 6; 5) 1.00 (purple), n = 12. Wildtype C57BL6/J mice were tested under non-targeted conditions. Prior to testing, animals were administered either ip Vehicle (**A**) or CGRP (0.1 mg/kg) (**B**). **C.** Using the asymptote value, light avoidance behavior is expressed as a function of log-spaced zone contrast across groups: 1) ip Vehicle (Veh, orange), 2) ip CGRP (teal), or 3) untreated (no ip injection) (black dotted line) from Figure 2 as a reference. Error bars indicate mean ± SEM.

**Figure S4. Effect of chronic intermittent administration of CGRP on light avoidance behavior to melanopsin stimulation on day 5. A.** Normalized time spent in the high contrast zone with the melanopsin as the targeted photoreceptor at a relative contrast level of 0.75. HLCKI mice were administered ip CGRP (0.1 mg/kg) or vehicle (Veh) on days 1, 3, 5, 7, and 9, with testing conducted on day 1 (see Figure 6A), day 5 (A), and day 9 (see Figure 6B). **C.** Asymptote value (AV) for each photoreceptor and genotype as outlined in panel A. For day 5, the groups tested included ip Veh (n = 22) and ip CGRP (n = 20). Each dot represents an individual animal. AV > 0 reflects light avoidance, and AV < 0 reflects light preference. Error bars indicate mean ± SEM.
